# Synthesis and Bioactivity Evaluation of *N*-Arylsulfonylindole Analogs Bearing a Rhodanine Moiety as Antibacterial Agents

**DOI:** 10.3390/molecules22060970

**Published:** 2017-06-14

**Authors:** Ming-Xia Song, Song-Hui Li, Jiao-Yang Peng, Ting-Ting Guo, Wen-Hui Xu, Shao-Feng Xiong, Xian-Qing Deng

**Affiliations:** Basic Medical and Pharmacy College, Jinggangshan University, Ji’an 343009, Jiangxi, China; freexiaoxiao83@aliyun.com (M.-X.S.); 18379698767@163.com (S.-H.L.); sakura367468@163.com (J.-Y.P.); m18379680319@163.com (T.-T.G.); 13687965920@163.com (W.-H.X.); 18370669382@163.com (S.-F.X.)

**Keywords:** rhodanine, *N*-arylsulfonylindole, propanoic acid, antibacterial activity

## Abstract

Due to the rapidly growing bacterial resistance to antibiotics and the scarcity of novel agents under development, bacterial infections are still a pressing global problem, making new types of antibacterial agents, which are effective both alone and in combination with traditional antibiotics, urgently needed. In this paper, seven series of *N*-arylsulfonylindole analogs **5**–**11** bearing rhodanine moieties were synthesized, characterized, and evaluated for antibacterial activity. According to the in vitro antimicrobial results, half of the synthesized compounds showed potent inhibition against four Gram-positive bacteria, with MIC values in the range of 0.5–8 µg/mL. For multidrug-resistant strains, compounds **6a** and **6c** were the most potent, with MIC values of 0.5 µg/mL, having comparable activity to gatifloxacin, moxiflocaxin and norfloxacin and being 128-fold more potent than oxacillin (MIC = 64 µg/mL) and 64-fold more active than penicillin (MIC = 32 µg/mL) against *Staphylococcus aureus* *ATCC 43300*.

## 1. Introduction

Bacterial drug resistance has emerged and become increasingly serious on a global scale, both in developed and developing countries [1]. Drug-resistant bacteria, such as methicillin-resistant *Staphylococcus aureus* (MRSA), multi-drug resistant *Escherichia coli*, and multi-drug resistant *Pseudomonas aeruginosa*, cause lethal diseases and cause great difficulties in the treatment of nosocomial infections [2,3,4,5], which severely threaten global public health while resulting in very large economic costs [6]. Currently the development of new antimicrobial drugs cannot keep pace with the development of bacterial drug resistance, and the number of new antibiotics approved for marketing per year is declining continuously. Taking advantage of pathogens virulence is an alternative strategy to inhibit antibiotic resistance development, and some studies have focused on anti-virulence agents against Gram-positive pathogens and Gram-negative pathogens [7,8]. However, this strategy has some weaknesses that limiting its development, such as the lack of a good way to assessing the efficacy and the inapplicability in immunocompromised patients. Over the last decade, a few new antibiotics (e.g., linezolid, ceftolozane, telavancin, ceftaroline fosamil, Xifaxan®, and daptomycin) were approved and dozens of antibiotics are currently in Phase 2 or Phase 3 clinical trials [9]. However, these drugs cannot address the entire spectrum of bacteria resistance [10], therefore, there is an urgent need to develop new antimicrobial agents, especially those with a new drug target or with the ability to overcome drug resistance.

Indole, an intercellular signaling molecule, regulates various aspects of bacterial physiology, including spore formation, plasmid stability, resistance to drugs, biofilm formation, and virulence. The amino acid tryptophan is an indole derivative and the precursor of the neurotransmitter serotonin. Up to now, indoles have displayed important physiological functions and potent pharmacological activities, including anti-inflammatory and antioxidant [11], antineoplastic [12,13,14], antimicrobial [15,16], antiviral [14,17,18], and anti-HIV activity [19,20]. *N*-Arylsulfonylindoles, as a kind of indole derivative, have received a great deal of attention in the field of chemical drug research, behaving as 5-HT_6_ receptor antagonists [21], anti-AIDS drugs [22], and antifungal agents [23]. However, the antibacterial activity of *N*-arylsulfonylindoles has not yet been reported to our knowledge.

**Scheme 1:**
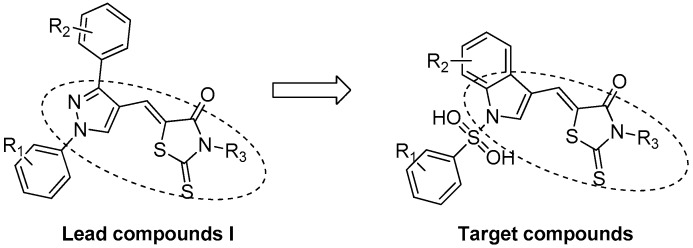
The design of the target compounds **5**–**11**.

Previously, we reported a series of rhodanine derivatives (compounds **I**, Scheme 1) which showed good inhibitory activity against Gram-positive bacteria (including multidrug-resistant clinical isolates) [24,25,26,27,28,29], which suggested that the rhodanine moiety was an important fragment for antimicrobial activity. Fragment-based drug discovery and skeleton migration strategy is a rational technique for molecular modification and drug design when some active fragments are obtained. Based upon these observations, and as part of our ongoing program aiming at the discovery and development of bioactive molecules, in this work, seven series of *N*-arylsulfonyl-3-substituted indoles **5**–**11** (Scheme 1) were designed using compound **I** as the lead compound. The target compounds were prepared by combining the rhodanine groups with *N*-arylsulfonylindoles, and their anti-bacterial activities were screened.

Chen et al. [30] have reported the synthesis and antibacterial activity evaluation of chalcone derivatives containing a rhodanine-3-acetic acid moiety, and found that the antibacterial activity of compounds with halogen and methyl substituents on the phenyl group was obviously higher than that of compounds with other substituents. In view of these findings, in this paper, only halogen and methyl substituents were chosen on the indole and phenyl sulfonyl moieties.

## 2. Results and Discussion

### 2.1. Chemistry

**Scheme 2:**
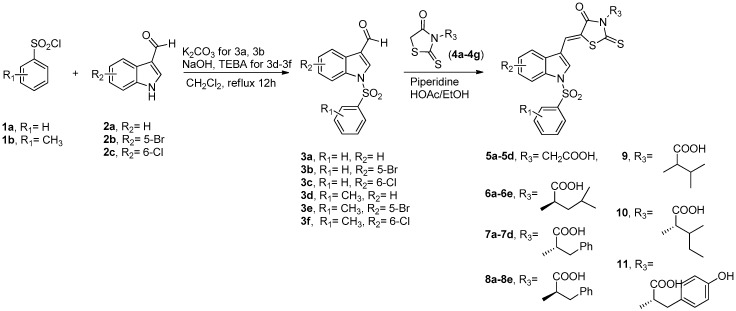
The synthesis route of compounds **5**–**11**.

The target compounds were synthesized as outlined in Scheme 2. A series of 3-substituted-5-((1-(phenylsulfonyl)-1*H*-indol-3-yl)methylene)-2-thioxothiazolidin-4-ones were synthesized using 1*H*-indole-3-carbaldehydes as the starting material. Firstly, benzenesulfonyl chlorides **1a**, **1b** were reacted with 1*H*-indole-3-carbaldehydes **2a**–**2c** in the presence of anhydrous potassium carbonate (K_2_CO_3_) at 40 °C to give 1-(phenylsulfonyl)-1*H*-indole-3-carbaldehydes **3a**–**f**, which were directly used in the next step without purification. Next compounds **3a**–**f** were subjected to a Knoevenagel condensation reaction with appropriate *N*-substituted rhodanines to provide seven new series of target compounds **5**–**11**. The structures of the products were well characterized by ^1^H-NMR, ^13^C-NMR, and high-resolution mass spectrometry.

### 2.2. Antimicrobial Activity

All of the target compounds **5**–**11** were evaluated for their in vitro anti-bacterial activity using a serial dilution method to obtain the minimum inhibitory concentration (MIC) against five Gram-positive strains (*S. aureus* (*CMCC(B) 26003* and *CMCC 25923*, *Streptococcus pyogenes CMCC 32067, Enterococcus faecalis CMCC 29212,* and *Bacillus subtilis CMCC 63501*), four Gram-negative strains (*E. coli* (*CMCC 25922* and *CMCC 44568*) and *P. aeruginosa* (*CMCC 27853* and *CMCC 10104*))m as well as two methicillin-resistant clinical isolates (*S. aureus ATCC 43300* and *ATCC 33591*). Gatifloxacin, moxifloxacin, norfloxacin, oxacillin, and penicillin were used as positive control drugs. 

Preliminarily, compounds **5**–**11** were screened for their activity against five Gram-positive strains and four Gram-negative strains. Initial screening results described as MIC values are presented in Table 1. The results illustrate that the inhibition of the seven series of derivatives against Gram-positive strains (effective against four bacteria) is in general superior to that of Gram-negative strains (effective against one bacterium). For Gram-positive strains, more than half of the tested compounds showed potent inhibition activity against *S. aureus (CMCC(B) 26003* and *CMCC 25923*), with MIC values in the range of 0.5–4 µg/mL. Half of the target compounds exhibited moderate activity against *E. faecalis CMCC 29212* (MIC = 4–32 µg/mL) and *S. pyogenes CMCC 32067* (MIC = 2–128 µg/mL). Conversely, five positive control agents did not exhibit inhibition activity for the two strains (MICs > 128 µg/mL). All compounds, however, had no effect on *B. subtilis CMCC 63501* at 128 µg/mL. For Gram-negative strains, only a few of compounds showed moderate activity against *P. aeruginosa CMCC 10104* with MICs of 4–64 µg/mL, while showing no inhibitory activity against three other strains at 128 µg/mL. The study found that, among the compounds synthesized, compound **8b** was the most active compound against two *S. aureus* with MIC values of 1 or 0.5 µg/mL, along with MIC = 2 or 4 µg/mL against *S. pyogenes CMCC 32067* and *E. faecalis CMCC 29212*, respectively. 

In the following trials, five compounds (**6a, 6c**, **8a–c**) were chosen to evaluate their inhibitory activity against two clinical isolates of multidrug-resistant Gram-positive bacterial strains (*S. aureus ATCC 43300* and *S. aureus ATCC 33591*), whose MICs against *S. aureus (CMCC(B) 26003* or *CMCC 25923* are less than 1 µg/mL. The results are listed in Table 2. The data illustrated that five compounds had excellent inhibitory activities against the two multidrug-resistant strains, with MICs of 0.5, 1, or 2 µg/mL. Among them, compounds **6a** and **6c** were the most potent, with MIC values of 0.5 µg/mL, having comparable activity to gatifloxacin, moxiflocaxin and norfloxacin, while being 128-fold more potent than oxacillin (MIC = 64 µg/mL) and 64-fold more active than penicillin (MIC = 32 µg/mL) against *S. aureus ATCC 43300*.

### 2.3. Cytotoxicity

The cytotoxic properties of compounds **6a**, **8b**, and **8c** were also investigated on HEK 293T cells using the CCK-8 method and the results are shown in Table 3. Compounds **6a**, **8b**, and **8c**, with IC_50_ values of 36.90, 54.09, and 32.28 µg/mL, respectively, were not cytotoxic at concentrations in the range of 0.5–16 µg/mL. The comparison between the MIC and IC_50_ values of the tested compounds suggests that compounds **6a**, **8b**, and **8c** exhibit in vitro antibacterial activity at non-cytotoxic concentrations. 

### 2.4. The Structure-Activity Relationships (SARs) Analysis

Based on the present data of the synthesized compounds, simple SARs could be proposed. For the derivatives bearing weak electron-donating substituents (R_1_ = -CH_3_), it seems that there is no obvious impact on the antibacterial activity in comparison with non-substituted compounds (R_1_ = H). In series **6**, for example, the MIC values against CMCC 26003 of compound **6c** (R_1_ = -CH_3_) and compound **6a** (R_1_ = H) were both 2 µg/mL, and the same result was discovered in the series **7**. As evidenced from Table 1, this could lead to the conclusion that a general inhibitory activity order of seven series of target compounds was series **6**, **8** > series **7**, **10** > series **9** > series **5**, **11**, but the differences are not remarkable. Upon comparison of series **7** and **8**, it can be found that the activity of the *R*-configuration compounds appears to be slightly better than that of *S*-configuration compounds.

## 3. Materials and Methods 

### 3.1. Instruments and Reagents

All of the reagents and solvents were purchased from Aladdin (Shanghai, China) or Sinopharm Chemical Reagent Co. Ltd. (Shanghai, China), and were used as received. Melting points were determined in open capillary tubes and are uncorrected. Reaction courses were monitored by thin-layer chromatography on silica gel-precoated F254 plates (Merck, Darmstadt, Germany). Developed plates were examined with UV lamps (254 nm). Nuclear magnetic resonance spectroscopy was performed on an AV-300 spectrometer (Bruker, Zurich, Switzerland) operating at 300 MHz for ^1^H and 75 MHz for ^13^C and using DMSO-*d*_6_ as solvent and tetramethylsilane as the internal standard. Electrospray Ionization Mass Spectrometry (ESI-MS) experiments were performed on an IT-TOF mass spectrometer (Shimadzu, Tokyo, Japan) in negative ion mode. Specific optical rotation was measured on a Digital automatic polariscope JASCO P-1020 (JASCO, Tokyo, Japan).

### 3.2. Synthesis Method and Spectral Data

#### 3.2.1. General Procedure for the Preparation of Compounds **3a**–**3b**

To a dry dichloromethane solution (10 mL) of the appropriate 1*H*-indole-3-carbaldehydes (2 mmol), anhydrous potassium carbonate (6 mmol) and benzenesulfonyl chlorides (4 mmol) in dry dichloromethane (20 mL) were added, and the mixture was stirred for 12 h at 40 °C. After the completion of the reaction, excess solvent was removed under reduced pressure to obtain a yellow crude solid of **3a**–**3b** which was directly used in the next step without purification. 

#### 3.2.2. General Procedure for the Preparation of Compounds **3c**–**3f**

To a solution of the appropriate 1*H*-indole-3-carbaldehydes (1 mmol) in dry dichloromethane (30 mL), sodium hydroxide (1.75 mmol), benzyltriethylammonium chloride (TEBA, 0.1 mmol), and benzenesulfonyl chlorides (1.2 mmol) were added and stirred for 12 h at room temperature. After the completion of the reaction, 15 mL water was added into the mixture. Then the mixture was extracted with dichloromethane (30 mL × 3). The combined organic layers were dried over anhydrous MgSO_4_ before being concentrated in vacuo. The crude products **3c**–**3f** obtained were directly used in the next step without purification. 

#### 3.2.3. General Procedure for the Preparation of Compounds **5**–**11**

A mixture of **3** (1 mmol), corresponding rhodanine (1 mmol), 10 drops glacial acetic acid and 10 drops piperidine in ethanol (20 mL) was refluxed for 16 h. After cooling, the solvent was evaporated in vacuo, followed by the purification of the resulting residue by silica gel column chromatography (dichloromethane/methanol = 100/1 or 150/1) to obtain a yellow solid **5**–**11***.*

#### 3.2.4. Spectral Data

*2-(5-((5-Bromo-1-(phenylsulfonyl)-1H-indol-3-yl)methylene)-4-oxo-2-thioxothiazolidin-3-yl)acetic acid* (**5a**). Yellow solid; yield 45%; m.p. 242–246 °C. ^1^H-NMR: δ 4.64 (s, 2H, NCH_2_), 7.65–8.38 (m, 10H, Ar-H, CH=C), 12.80 (br.s, 1H, COOH). ^13^C-NMR: *δ* 192.57, 167.35, 166.27, 136.47, 135.98, 133.12, 131.05, 130.66, 129.50, 129.00, 127.79, 127.75, 125.95, 123.79, 117.92, 116.80, 115.59, 53.18. ESI-HRMS calcd. for C_20_H_12_BrN_2_O_5_S_3_^−^ ([M − H]^−^): 534.9097; found: 534.9118.

*2-(5-((6-Chloro-1-(phenylsulfonyl)-1H-indol-3-yl)methylene)-4-oxo-2-thioxothiazolidin-3-yl)acetic acid* (**5b**). Yellow solid; yield 47%; m.p. 206–208 °C. ^1^H-NMR: δ 4.40 (s, 2H, NCH_2_), 7.44–8.24 (m, 10H, Ar-H, CH=C), 9.24 (br.s, 1H, COOH). ^13^C-NMR: *δ* 192.54, 167.13, 166.62, 136.53, 136.02, 134.67, 131.51, 130.75, 128.35, 127.99, 127.76, 125.39, 124.67, 122.56, 121.59, 117.33, 113.31, 43.98. ESI-HRMS calcd. for C_20_H_12_ClN_2_O_5_S_3_^−^ ([M − H]^−^): 490.9602; found: 490.9615.

*2-(5-((5-Bromo-1-tosyl-1H-indol-3-yl)methylene)-4-oxo-2-thioxothiazolidin-3-yl)acetic acid* (**5c**). Yellow solid; yield 44%; m.p. 226 °C. ^1^H-NMR: δ 2.33 (s, 3H, CH_3_), 4.40 (s, 2H, NCH_2_), 7.37–8.40 (m, 9H, Ar-H, CH=C), 9.31 (br.s, 1H, COOH). ^13^C-NMR: *δ* 194.93, 178.62, 176.66, 147.06, 146.25, 133.97, 133.13, 132.73, 132.63, 131.08, 130.76, 128.74, 127.75, 127.53, 117.81, 116.94, 115.62, 43.85, 22.70. ESI-HRMS calcd. for C_21_H_14_BrN_2_O_5_S_3_^−^ ([M − H]^−^): 548.9254; found: 548.9272.

*2-(5-((6-Chloro-1-tosyl-1H-indol-3-yl)methylene)-4-oxo-2-thioxothiazolidin-3-yl)acetic acid* (**5d**). Yellow solid; yield 57%; m.p. 205 °C. ^1^H-NMR: δ 2.35 (s, 3H, CH_3_), 4.41 (s, 2H, NCH_2_), 7.42–8.11 (m, 9H, Ar-H, CH=C), 9.28 (br.s, 1H, COOH). ^13^C-NMR: *δ* 192.52, 167.45, 166.62, 147.13, 134.63, 133.57, 131.44, 131.16, 128.36, 127.96, 127.79, 125.29, 124.48, 122.46, 121.63, 117.15, 113.30, 43.89, 22.73. ESI-HRMS calcd. for C_21_H_14_ClN_2_O_5_S_3_^−^ ([M − H]^−^): 504.9759; found: 504.9774.

*(R)-2-(5-((1-(Phenylsulfonyl)-1H-indol-3-yl)methylene)-4-oxo-2-thioxothiazolidin-3-yl)-4-methylpentanoic acid* (**6a**). Yellow solid; yield 51%; m.p. 271–276 °C. ${\left[\alpha \right]}_{D}^{20}$: +35 (c = 0.20, DMF). ^1^H-NMR: δ 0.89 (d, 3H, *J* = 6.4 Hz, CHCH_3_), 0.94 (d, 3H, *J* = 6.4 Hz, CHCH_3_), 1.35–1.45 (m, 1H, CHCH_3_), 1.99–2.07 (m, 1H, CH-H_a_), 2.20–2.28 (m, 1H, CH-H_b_), 5.61 (br.s, 1H, NCH), 7.38–8.20 (m, 11H, Ar-H, CH=C), 13.31 (br.s, 1H, COOH). ^13^C-NMR: *δ* 193.05, 169.83, 166.48, 136.66, 135.80, 134.29, 130.56, 129.03, 128.46, 127.72, 126.88, 125.03, 123.82, 122.02, 120.82, 117.21, 113.72, 56.47, 36.88, 25.29, 23.36, 22.37. ESI-HRMS calcd. for C_24_H_21_N_2_O_5_S_3_^−^([M − H]^−^): 513.0618; found: 513.0629.

*(R)-2-(5-((6-Chloro-1-(phenylsulfonyl)-1H-indol-3-yl)methylene)-4-oxo-2-thioxothiazolidin-3-yl)-4-methyl-pentanoic acid* (**6b**). Yellow solid; yield 53%; m.p. 180–181 °C. ${\left[\alpha \right]}_{D}^{20}$: +62.5 (c = 0.20, DMF). ^1^H-NMR: δ 0.86 (d, 3H, *J* = 6.6 Hz, CHCH_3_), 0.91 (d, 3H, *J* = 6.6 Hz, CHCH_3_), 1.33–1.38 (m, 1H, CHCH_3_), 2.00–2.07 (m, 1H, CH-H_a_), 2.24–2.31 (m, 1H, CH-H_b_), 5.50 (br.s, 1H, NCH), 7.20–8.22 (m, 10H, Ar-H, CH=C), 9.30 (br.s, 1H, COOH). ^13^C-NMR: *δ* 193.22, 170.01, 166.56, 136.51, 136.00, 134.63, 131.53, 130.70, 129.78, 128.10, 127.76, 125.94, 125.38, 122.47, 120.63, 117.16, 113.29, 56.50, 37.23, 25.57, 23.49, 22.68. ESI-HRMS calcd. for C_24_H_20_ClN_2_O_5_S_3_^−^ ([M − H]^−^): 547.0228; found: 547.0244.

*(R)-2-(5-((1-Tosyl-1H-indol-3-yl)methylene)-4-oxo-2-thioxothiazolidin-3-yl)-4-methylpentanoic acid* (**6c**). Yellow solid; yield 66%; m.p. 200 °C. ${\left[\alpha \right]}_{D}^{20}$ +45 (c = 0.10, DMF). ^1^H-NMR: δ 0.88 (d, 3H, *J* = 6.4 Hz, CHCH_3_), 0.93 (d, 3H, *J* = 6.5 Hz, CHCH_3_), 1.28–1.33 (m, 1H, CHCH_3_), 1.97–2.07 (m, 1H, CH-H_a_), 2.20–2.27 (m, 1H, CH-H_b_), 2.33 (s, 3H, Ph-CH_3_), 5.61 (br.s, 1H, NCH), 7.37–7.51 (m, 4H, Ar-H), 7.98–8.07 (m, 6H, Ar-H, CH=C), 13.04 (br.s, 1H, COOH). ^13^C-NMR: *δ* 193.01, 171.68, 169.83, 157.49, 146.85, 134.27, 133.73, 130.97, 129.02, 128.48, 127.76, 126.80, 124.95, 123.87, 120.75, 117.05, 113.74, 56.46, 36.88, 25.82, 23.36, 22.37, 21.54. ESI-HRMS calcd. for C_25_H_23_N_2_O_5_S_3_^−^ ([M − H]^−^): 527.0775; found: 527.0790.

*(R)-2-(5-((5-Bromo-1-tosyl-1H-indol-3-yl)methylene)-4-oxo-2-thioxothiazolidin-3-yl)-4-methylpentanoic acid* (**6d**). Yellow solid; yield 61%; m.p. 256–260 °C. ${\left[\alpha \right]}_{D}^{20}$: +57.5 (c = 0.08, DMF). ^1^H-NMR: δ 0.89 (d, 3H, *J* = 6.3 Hz, CHCH_3_), 0.94 (d, 3H, *J* = 6.3 Hz, CHCH_3_), 1.45–1.53 (m, 1H, CHCH_3_), 1.99–2.08 (m, 1H, CH-H_a_), 2.20–2.26 (m, 1H, CH-H_b_), 2.34 (s, 3H, Ph-CH_3_), 5.62 (br.s, 1H, NCH), 7.43 (d, 2H, *J* = 7.9 Hz, Ph-H), 7.61 (d, 1H, *J* = 8.7 Hz, Ph-H), 7.93–8.07 (m, 5H, Ar-H), 8.37 (s, 1H, CH=C), 13.23 (br.s, 1H, COOH). ^13^C-NMR: *δ* 193.01, 171.68, 169.83, 157.49, 146.85, 134.27, 133.73, 130.97, 129.02, 128.48, 127.76, 126.80, 124.95, 123.87, 120.75, 117.05, 113.74, 56.46, 36.88, 25.82, 23.36, 22.37, 21.54. ESI-HRMS calcd. for C_25_H_22_BrN_2_O_5_S_3_^−^ ([M − H]^−^): 604.9880; found: 604.9899.

*(R)-2-(5-((6-Chloro-1-tosyl-1H-indol-3-yl)methylene)-4-oxo-2-thioxothiazolidin-3-yl)-4-methylpentanoic acid* (**6e**). Yellow solid; yield 63%; m.p. 247–249 °C. ${\left[\alpha \right]}_{D}^{20}$: +31.5 (c = 0.20, DMF). ^1^H-NMR: δ 0.88 (d, 3H, *J* = 6.6 Hz, CHCH_3_), 0.94 (d, 3H, *J* = 6.6 Hz, CHCH_3_), 1.46–1.54 (m, 1H, CHCH_3_), 1.97–2.07 (m, 1H, CH-H_a_), 2.21–2.28 (m, 1H, CH-H_b_), 2.34 (s, 3H, Ph-CH_3_), 5.60 (br.s, 1H, NCH), 7.45 (d, 3H, *J* = 8.3 Hz, Ar-H), 7.97–8.11 (m, 6H, Ar-H, CH=C), 13.04 (br.s, 1H, COOH). ^13^C-NMR: *δ* 193.01, 169.80, 166.44, 147.16, 134.61, 133.54, 131.50, 131.13, 129.00, 127.88, 127.84, 125.34, 123.48, 122.51, 122.42, 116.93, 113.30, 56.46, 36.86, 25.28, 23.36, 22.36, 21.58. ESI-HRMS calcd. for C_25_H_22_ClN_2_O_5_S_3_^−^ ([M − H]^−^): 561.0385; found: 561.0406.

*(S)-2-(4-Oxo-5-((1-(phenylsulfonyl)-1H-indol-3-yl)methylene)-2-thioxothiazolidin-3-yl)-3-phenylpropanoic acid* (**7a**). Yellow solid; yield 58%; m.p. 156–157 °C. ${\left[\alpha \right]}_{D}^{20}$: –150 (c = 0.12, DMF). ^1^H-NMR: δ 3.56 (d, 2H, *J* = 7.6 Hz, CHCH_2_), 5.76 (br.s, 1H, NCH), 7.15–8.19 (m, 16H, Ar-H, CH=C), 8.85 (br.s, 1H, COOH). ^13^C-NMR: *δ* 192.67, 169.05, 166.55, 138.07, 136.66, 135.78, 134.23, 130.57, 129.32, 129.03, 128.73, 128.20, 127.74, 126.93, 126.85, 124.99, 122.82, 122.17, 120.77, 117.11, 113.70, 44.05, 22.70. ESI-HRMS calcd. for C_27_H_19_N_2_O_5_S_3_^−^ ([M − H]^−^): 547.0462; found: 547.0491.

*(S)-2-(5-((6-Chloro-1-(phenylsulfonyl)-1H-indol-3-yl)methylene)-4-oxo-2-thioxothiazolidin-3-yl)-3-phenyl-propanoic acid* (**7b**). Yellow solid; yield 54%; m.p. 220–224 °C. ${\left[\alpha \right]}_{D}^{20}$: –144.5 (c = 0.40, DMF). ^1^H-NMR: δ 3.54 (br.s, 2H, CHCH_2_), 5.91 (br.s, 1H, NCH), 7.19–8.24 (m, 15H, Ar-H, CH=C), 13.26 (br.s, 1H, COOH). ^13^C-NMR: *δ* 192.36, 169.17, 166.37, 137.05, 136.49, 136.03, 134.56, 131.54, 130.70, 129.83, 129.45, 128.93, 128.78, 128.47, 127.85, 127.20, 125.39, 123.23, 122.52, 116.87, 113.25, 44.85, 25.77. ESI-HRMS calcd. for C_27_H_18_ClN_2_O_5_S_3_^−^ ([M − H]^−^): 581.0072; found: 581.0094.

*(S)-2-(5-((1-Tosyl-1H-indol-3-yl)methylene)-4-oxo-2-thioxothiazolidin-3-yl)-3-phenylpropanoic acid* (**7c**). Yellow solid; m.p. 164–166 °C, yield 55%. ${\left[\alpha \right]}_{D}^{20}$: –174.25 (c = 0.40, DMF). ^1^H-NMR: δ 2.32 (s, 3H, Ph-CH_3_), 3.56 (d, 2H, *J* = 7.0 Hz, CHCH_2_), 5.71 (br.s, 1H, NCH), 7.14–7.50 (m, 9H, Ar-H), 7.90–8.05 (m, 6H, Ar-H, CH=C), 9.10 (br.s, 1H, COOH). ^13^C-NMR: *δ* 192.81, 169.00, 166.64, 146.83, 138.35, 134.21, 133.72, 130.98, 129.27, 129.04, 128.70, 128.13, 127.77, 126.84, 126.77, 124.90, 122.62, 122.16, 120.71, 116.99, 113.70, 43.94, 22.70, 21.55. ESI-HRMS calcd. for C_28_H_21_N_2_O_5_S_3_^−^ ([M − H]^−^): 561.0618; found: 561.06392.

*(S)-2-(5-((5-Bromo-1-tosyl-1H-indol-3-yl)methylene)-4-oxo-2-thioxothiazolidin-3-yl)-3-phenylpropanoic acid* (**7d**). Yellow solid; yield 59%; m.p.217–221 °C. ${\left[\alpha \right]}_{D}^{20}$: –216.25 (c = 0.08, DMF). ^1^H-NMR: δ 2.34 (s, 3H, Ph-CH_3_), 3.56 (d, 2H, *J* = 6.8 Hz, CHCH_2_), 5.65 (br.s, 1H, NCH), 7.15–7.63 (m, 9H, Ar-H), 7.86–8.05 (m, 4H, Ar-H), 8.35 (s, 1H, CH=C). ^13^C-NMR: *δ* 191.51, 173.04, 170.18, 148.15, 146.96, 142.46, 133.52, 133.24, 133.06, 131.08, 131.04, 129.33, 129.23, 128.68, 127.92, 127.80, 126.93, 126.82, 121.1, 117.9, 113.5, 44.13, 22.72, 22.17. ESI-HRMS calcd. for C_28_H_2__0_BrN_2_O_5_S_3_^−^ ([M − H]^−^): 638.9723; found: 638.9745.

*(R)-2-(4-Oxo-5-((1-(phenylsulfonyl)-1H-indol-3-yl)methylene)-2-thioxothiazolidin-3-yl)-3-phenylpropanoic acid* (**8a**). Yellow solid; yield 65%; m.p. 198–199 °C. ${\left[\alpha \right]}_{D}^{20}$: +255 (c = 0.02, DMF). ^1^H-NMR: δ 3.57 (d, 2H, *J* = 6.8 Hz, CHCH_2_), 5.63 (br.s, 1H, NCH), 7.13–8.17 (m, 16H, Ar-H, CH=C), 9.26 (br.s, 1H, COOH). ^13^C-NMR: *δ* 192.92, 169.15, 166.77, 139.01, 136.66, 135.76, 134.23, 130.56, 129.17, 129.07, 128.66, 127.93, 127.71, 126.82, 126.66, 124.97, 122.65, 121.98, 120.73, 117.21, 113.69, 43.89, 22.71. ESI-HRMS calcd. for C_27_H_19_N_2_O_5_S_3_^−^ ([M − H]^−^): 547.0462; found: 547.0487.

*(R)-2-(5-((6-Chloro-1-(phenylsulfonyl)-1H-indol-3-yl)methylene)-4-oxo-2-thioxothiazolidin-3-yl)-3-phenyl-propanoic acid* (**8b**). Yellow solid; yield 61%; m.p. 264–265 °C. ${\left[\alpha \right]}_{D}^{20}$: +170.36 (c = 0.28, DMF). ^1^H-NMR: δ 3.54 (d, 2H, *J* = 6.9 CHCH_2_), 5.91 (br.s, 1H, NCH), 7.20–8.23 (m, 15H, Ar-H, CH=C), 12.77 (br.s, 1H, COOH). ^13^C-NMR: *δ* 192.35, 169.15, 166.35, 137.00, 136.48, 136.04, 134.57, 131.55, 130.71, 129.45, 128.96, 128.79, 127.85, 127.21, 125.39, 123.29, 122.54, 122.25, 116.86, 113.26, 100.00, 58.76, 29.48. ESI-HRMS calcd. for C_27_H_18_ClN_2_O_5_S_3_^−^ ([M − H]^−^): 581.0072; found: 581.0090.

*(R)-2-(5-((1-Tosyl-1H-indol-3-yl)methylene)-4-oxo-2-thioxothiazolidin-3-yl)-3-phenylpropanoic acid* (**8c**). Yellow solid; yield 57%; m.p. 176–181 °C. ${\left[\alpha \right]}_{D}^{20}$: +178.25 (c = 0.40, DMF). ^1^H-NMR: δ 2.32 (s, 3H, Ph-CH_3_), 3.57 (d, 2H, *J* = 7.1 Hz, CHCH_2_), 5.71 (br.s, 1H, NCH), 7.14–7.50 (m, 9H, Ar-H), 7.90–8.05 (m, 6H, Ar-H, CH=C), 9.16 (br.s, 1H, COOH). ^13^C-NMR: *δ* 191.80, 169.17, 166.65, 146.82, 138.37, 134.21, 133.73, 130.97, 129.26, 129.04, 128.69, 128.12, 127.76, 126.83, 126.76, 124.89, 122.58, 122.17, 120.70, 116.99, 113.70, 43.90, 22.70, 21.54. ESI-HRMS calcd. for C_28_H_21_N_2_O_5_S_3_^−^ ([M − H]^−^): 561.0618; found: 561.0643.

*(R)-2-(5-((5-Bromo-1-tosyl-1H-indol-3-yl)methylene)-4-oxo-2-thioxothiazolidin-3-yl)-3-phenylpropanoic acid* (**8d**). Yellow solid; yield 62%; m.p. 192–193 °C. ${\left[\alpha \right]}_{D}^{20}$: +159.13 (c = 0.80, DMF). ^1^H-NMR: δ 2.34 (s, 3H, Ph-CH_3_), 3.56 (d, 2H, *J* = 6.5 Hz, CHCH_2_), 5.71 (br.s, 1H, NCH), 7.13–7.20 (m, 5H, Ar-H), 7.43 (d, 2H, *J* = 8.3 Hz, Ar-H), 7.61 (dd, 1H, *J*_1_ = 8.9 Hz, *J*_2_ = 1.8 Hz, Ar-H), 7.91–8.06 (m, 5H, Ar-H), 8.37 (s, 1H, CH=C). ^13^C-NMR: *δ* 192.71, 169.11, 166.51, 147.05, 138.38, 138.27, 133.52, 133.05, 131.04, 129.39, 129.27, 129.01, 128.70, 127.80, 126.86, 123.67, 122.62, 122.51, 117.80, 116.53, 115.57, 43.94, 22.71, 21.56. ESI-HRMS calcd. for C_28_H_20_BrN_2_O_5_S_3_^−^ ([M − H]^−^): 638.9723; found: 638.9740.

*(R)-2-(5-((6-Chloro-1-tosyl-1H-indol-3-yl)methylene)-4-oxo-2-thioxothiazolidin-3-yl)-3-phenylpropanoic acid (***8e**). Yellow solid; yield 55%; m.p. 277–280 °C. ${\left[\alpha \right]}_{D}^{20}$: +182.5 (c = 0.80, DMF). ^1^H-NMR: δ 2.34 (s, 3H, Ph-CH_3_), 3.56 (d, 2H, *J* = 7.0 Hz, CHCH_2_), 5.84 (br.s, 1H, NCH), 7.18–7.45 (m, 8H, Ar-H), 7.92–8.10 (m, 6H, Ar-H). ^13^C-NMR: *δ* 192.32, 169.23, 166.43, 147.14, 137.43, 134.54, 133.53, 131.46, 131.12, 129.38, 128.88, 128.76, 127.87, 127.09, 125.30, 122.97, 122.49, 122.34, 116.76, 113.26, 110.60, 33.77, 29.48, 21.58. ESI-HRMS calcd. for C_28_H_2__0_ClN_2_O_5_S_3_^−^ ([M − H]^−^): 595.0228; found: 595.0245.

*2-(4-Oxo-5-((1-(phenylsulfonyl)-1H-indol-3-yl)methylene)-2-thioxothiazolidin-3-yl)3-methylbutanoic acid (***9**). Yellow solid; yield 46%; m.p. 255–257 °C. ^1^H-NMR: δ 0.78 (d, 3H, *J* = 5.8 Hz, CHCH_3_), 1.22 (d, 3H, *J* = 4.6 Hz, CHCH_3_), 2.77 (d, 1H, CH(CH_3_)_2_), 5.21 (d, 1H, *J* = 7.5 Hz, NCH), 7.41–8.20 (m, 11H, Ar-H, CH=C), 13.15 (br.s, 1H, COOH). ^13^C-NMR: *δ* 193.02, 169.09, 166.53, 136.67, 135.81, 134.29, 130.57, 129.03, 128.57, 127.76, 126.90, 125.04, 124.16, 121.71, 120.83, 117.17, 113.73, 62.69, 27.65, 22.15, 19.41. ESI-HRMS calcd. for C_23_H_19_N_2_O_5_S_3_^−^ ([M − H]^−^): 499.0462; found: 499.0479.

*(S)-2-(4-Oxo-5-((1-(phenylsulfonyl)-1H-indol-3-yl)methylene)-2-thioxothiazolidin-3-yl)-3-methylpentanoic acid* (**10**). Yellow solid; yield 42%; m.p. 256–260 °C. ${\left[\alpha \right]}_{D}^{20}$: –32.5 (c = 0.4, CHCl_3_). ^1^H-NMR: δ 0.82 (t, 3H, *J* = 7.2 Hz, CH_2_CH_3_), 0.94–1.01 (m, 1H, CH-H_a_), 1.18 (d, 3H, *J* = 6.5 Hz, CHCH_3_), 1.47–1.53 (m, 1H, CH-H_b_), 2.54–2.59 (m, 1H, CHCH_3_), 5.25 (d, 1H, *J* = 7.4 Hz, NCH), 7.39–8.20 (m, 11H, Ar-H, CH=C), 13.18 (br.s, 1H, COOH). ^13^C-NMR: *δ* 193.00, 169.09, 166.57, 136.65, 135.81, 134.27, 130.57, 129.02, 128.58, 127.75, 126.89, 125.03, 124.19, 121.66, 120.84, 117.17, 113.71, 62.15, 33.53, 25.37, 18.05, 11.34. ESI-HRMS calcd. for C_24_H_21_N_2_O_5_S_3_^−^ ([M − H]^−^): 513.0629; found: 513.0618.

*(S)-3-(4-Hydroxyphenyl)-2-(4-oxo-5-((1-(phenylsulfonyl)-1H-indol-3-yl)methylene)-2-thioxothiazolidin-3-yl)propanoic acid (***11**). Yellow solid; yield 47%; m.p. 167–181 °C. ${\left[\alpha \right]}_{D}^{20}$: –150 (c = 0.2, DMF). ^1^H-NMR: δ 3.46 (br.s, 2H, CHCH_2_), 5.69 (br.s, 1H, NCH), 6.59 (d, 2H, *J* = 8.1 Hz, Ar-H), 6.94 (d, 2H, *J* = 8.1 Hz, Ar-H), 7.38–8.19 (m, 11H, Ar-H, CH=C), 9.13 (s, 1H, OH). ^13^C-NMR: *δ* 192.67, 170.93, 166.57, 156.25, 136.66, 135.77, 134.22, 130.70, 130.56, 130.18, 129.05, 128.69, 128.16, 127.95, 127.75, 126.83, 124.98, 120.75, 117.10, 115.57, 113.69, 44.84, 25.78. ESI-HRMS calcd. for C_27_H_19_N_2_O_6_S_3_^−^ ([M − H]^−^): 563.0411; found: 563.0430.

### 3.3. Evaluation of Anti-Bacterial Activity In Vitro

The anti-bacterial activity in vitro against *S. aureus* (CMCC(B) 26003 and CMCC 25923, *S. pyogenes* CMCC 32067, *E. faecalis* CMCC 29212, *B. subtilis* CMCC 63501; *E. coli* CMCC 25922 and CMCC 44568, *P. aeruginosa* CMCC 27853 and CMCC 10104, as well as two methicillin-resistant clinical isolates (*S. aureus* ATCC 43300 and ATCC 33591) was evaluated using a two-fold serial dilution technique [31], and the final concentrations of compounds obtained were in the range of 0.5–128 μg/mL. Test bacteria were grown to mid-log phase in Mueller-Hinton broth (MHB) or Tryptone Soya Broth (TSB) and diluted 1000-fold in the same medium. The bacteria of 10^5^ CFU/mL were inoculated into MHB or TSB and dispensed at 0.2 mL/well in a 96-well microtiter plate. As positive controls, gatifloxacin, moxifloxacin, norfloxacin, oxacillin, and penicillin were used. Test compounds were prepared in DMSO, the final concentration of which did not exceed 0.05%. The MIC was defined as the concentration of a test compound that completely inhibited bacteria growth during 24 h incubation at 37 °C. Bacteria growth was determined by measuring the absorption at 630 nm using a microtiter enzyme-linked immunosorbent assay (ELISA) reader. All experiments were carried out three times.

### 3.4. Evaluation of Cytotoxicity In Vitro 

HEK 293T cells were used to test the cytotoxicity of the new compounds. HEK 293T cells were grown in Dulbecco modified Eagle medium supplemented with fetal bovine serum (10%), and antibiotics (penicillin-streptomycin mixture (100 U/mL)). Cells at 80% to 90% confluence were split by trypsin (0.25% in PBS; pH 7.4), and the medium was changed at 24 h intervals. The cells were cultured at 37 °C in a 5% CO_2_ incubator. The cells were grown to three passages, and approximately 1 × 10^4^ cells were seeded into each well of a 96-well plate and allowed to incubate to allow attachment of the cells to the substrate. After 24 h, the medium was replaced with DMEM supplemented with 10% FBS containing various concentrations (4, 8, 16, 32, 64, 128 μg/mL) of test compounds and incubated for 48 h. Each concentration set three wells in parallel. Then 20 µL of CCK-8 solution was added to each well. After incubation for 3 h, the optical density was measured at 450 nm using a microtiter ELISA reader. The IC_50_ values were defined as the concentrations inhibiting 50% of cell growth. 

## 4. Conclusions

In summary, seven new series of *N*-arylsulfonylindoles **5**–**11** bearing rhodanine moieties were designed, synthesized, and evaluated for their antibacterial activities. In accordance to the results of antibacterial tests in vitro, some of the compounds showed good antibacterial activities against *Staphylococcus aureus*, including multidrug-resistant strains. Among them compounds **6a** and **6c** showed the most potent levels of activity (MIC = 0.5 µg/mL) against selected MRSA strains. These results illustrate that *N*-arylsulfonylindole analogs bearing rhodanine moieties are promising leads to develop novel antimicrobial agents against many infections caused by Gram-positive strains, especially *Staphylococcus aureus*, incluing MRSA. Future studies will focus on the mechanism of action of these compounds.

## Figures and Tables

**Table 1:**
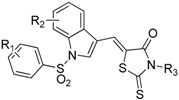
Inhibitory activity (MIC, μg/mL) of compounds **5**–**11** against Gram-positive and Gram-negative bacteria.

Compd.	R_1_-	R_2_-	R_3_-	Gram-Positive Strains					Gram-Negative Strains			
				26003 ^a^	25923 ^b^	32067 ^c^	29212 ^d^	63501 ^e^	25922 ^f^	44568 ^g^	27853 ^h^	10104 ^i^
**5a**	H	5-Br	CH_2_COOH	8	8	>128	32	>128	>128	>128	>128	32
**5b**	H	6-Cl	CH_2_COOH	>128	>128	>128	>128	>128	>128	>128	>128	>128
**5c**	CH_3_	5-Br	CH_2_COOH	8	16	>128	>128	>128	>128	>128	>128	>128
**5d**	CH_3_	6-Cl	CH_2_COOH	4	4	>128	32	>128	>128	>128	>128	32
**6a**	H	H	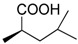	2	1	16	8	>128	>128	>128	>128	8
**6b**	H	6-Cl	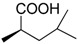	4	2	>128	16	>128	>128	>128	>128	16
**6c**	CH_3_	H	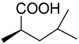	2	1	>128	4	>128	>128	>128	>128	4
**6d**	CH_3_	5-Br	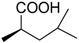	2	2	>128	4	>128	>128	>128	>128	4
**6e**	CH_3_	6-Cl	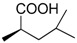	64	>128	>128	>128	>128	>128	>128	>128	>128
**7a**	H	H	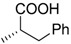	2	2	128	4	>128	>128	>128	>128	4
**7b**	H	6-Cl	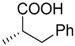	32	16	>128	8	>128	>128	>128	>128	8
**7c**	CH_3_	H	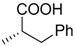	2	16	>128	>128	>128	>128	>128	>128	>128
**7d**	CH_3_	5-Br	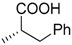	2	2	>128	16	>128	>128	>128	>128	16
**8a**	H	H	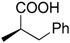	2	1	4	16	>128	>128	>128	>128	16
**8b**	H	6-Cl	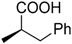	1	0.5	2	4	>128	>128	>128	>128	4
**8c**	CH_3_	H	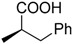	1	1	128	>128	>128	>128	>128	>128	>128
**8d**	CH_3_	5-Br	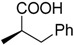	2	2	>128	64	>128	>128	>128	>128	64
**8e**	CH_3_	6-Cl	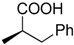	2	2	>128	64	>128	>128	>128	>128	64
**9**	H	H	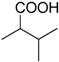	4	4	>128	32	>128	>128	>128	>128	32
**10**	H	H	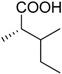	2	2	16	16	>128	>128	>128	>128	16
**11**	H	H	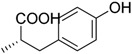	>128	8	>128	32	>128	>128	>128	>128	32
**Gatifloxacin**	—	—	—	0.125	0.125	>128	>128	1	2	0.125	0.125	2
**Moxifloxacin**	—	—	—	0.125	0.125	>128	>128	1	2	0.125	0.125	2
**Norfloxacin**	—	—	—	0.125	0.125	>128	>128	1	2	0.125	0.125	2
**Oxacillin**	—	—	—	0.125	0.125	>128	>128	128	>128	128	>128	>128
**penicillin**	—	—	—	0.125	0.125	>128	>128	128	128	128	>128	>128

^a^ *S. aureus* CMCC(B) 26003; ^b^ *S. aureus* CMCC 25923; ^c^ *S. pyogenes* CMCC 32067; ^d^ *E. faecalis* CMCC 29212; ^e^ *B. subtilis* CMCC 63501; ^f^ *E. coli* CMCC 25922; ^g^ *E. coli* CMCC 44568; ^h^ *P*. *aeruginosa* CMCC 27853; ^I^ *P. aeruginosa* CMCC 10104.

**Table 2:** Inhibitory activity (MIC, µg/mL) of compounds **6a**, **6c** and **8a**–**c** against clinical isolates of multidrug-resistant strains.

Compound	R_1_-	R_2_-	R_3_-	Multidrug-Resistant Gram-Positive Strains	
				43300 ^a^	33591 ^b^
**6a**	H	H	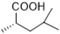	0.5	1
**6c**	CH_3_	H	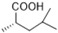	0.5	1
**8a**	H	H	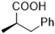	1	2
**8b**	H	6-Cl	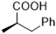	2	2
**8c**	CH_3_	H	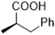	1	2
**Gatifloxacin**	—	—	—	0.5	0.25
**Moxifloxaci** **n**	—	—	—	0.5	0.25
**Norfloxacin**	—	—	—	0.5	0.25
**Oxacillin**	—	—	—	64	8
**Penicillin**	—	—	—	32	>32

^a^ *S. aureus* ATCC 43300; ^b^ *S. aureus* ATCC 33591.

**Table 3:** Cytotoxic activity of compounds **6a, 8b** and **8c** on the HEK 293T cells.

Compound	IC_50_ (µg/mL) ^a^
**6a**	36.90
**8b**	54.09
**8c**	32.28

^a^ IC_50_ is the concentration required to inhibit 50% of cell growth.
